# Personal

**Published:** 1915-05

**Authors:** 


### PERSONAL.

---

**Announcement of removal, travel, and other matters of interest are requested. Please report errors in the listing of any physician in the State and other directories, that we may co-operate with the State Society in securing a correct list.**

---

Dr. F. M. O'Gorman of Buffalo visited Pittsburg, Philadelphia and Atlantic City, in April.

---

Dr. Charles J. Mengis of Buffalo has returned from California.

---

The automobile of Dr. Edward J. Cook of Buffalo was stolen on the evening of April 5. It was found deserted but not damaged, early the next morning.

---

Dr. Robert W. Hinds of Buffalo, sailed for service with the Red Cross, April 16.

---

Dr. Allen Wheeler Holmes announces his association with the Jackson Health Resort of Dansville, N. Y.

---

Dr. Guy Cluxton Boughton of Erie, announces that he has secured a supply of radium for therapeutic purposes.

---

Dr. Burt C. Johnson of Buffalo announces the removal of his office to 1355 Main Street, corner of Laurel Street, May 1.

---

Dr. Wm. M. Sill of Jamestown was taken sick with smallpox on the evening of April 8, while attending a concert. He was exposed to a case in Ellery, a few weeks before. He had been vaccinated in childhood but not since, till a week after exposure. His infection is, however, very mild.

---

Dr. Wm. G. Ring of Buffalo has moved to 34 St. James Place.

Dr. Walter C. Miller, who has been in the service of the U. S. bureau of food and drug inspection, at Buffalo, for eight years, has been transferred to the N. Y. office and has been succeeded by Dr. O. R. Sudler formerly of Baltimore.

---

Dr. Percy C. Cripps of Buffalo is traveling in North Carolina.

